# A case report of wandering spleen with pedicle torsion and splenic infarction being misdiagnosed as organ inversion complicated with acute appendicitis

**DOI:** 10.3389/fsurg.2022.916426

**Published:** 2022-09-06

**Authors:** Shengjie Zhao, Yindi Wang, Zhiheng Wan, Hancheng Chen, Xinyu Zhao, Ruibin Li

**Affiliations:** ^+^ Ambulatory Surgery Center, First Affiliated Hospital of Baotou Medical College, Inner Mongolia University of Science and Technology, Baotou, China

**Keywords:** wandering spleen, pedicle torsion, acute abdomen, diagnosis, treatment

## Abstract

Wandering spleen is a rare disease that is easily misdiagnosed. When combined with splenic pedicle torsion and even splenic infarction, wandering spleen is a rare and critical cause of surgical acute abdomen. We report an 18-year-old male patient with abdominal organ inversion diagnosed as acute appendicitis before operation. Laparoscopic exploration confirmed wandering spleen with splenic pedicle torsion led to splenic infarction and was complicated by appendicitis. He was treated with laparoscopic appendectomy and abdominal splenectomy. The patient recovered well after the operation and was discharged from the hospital in 7 days. During the 4-year follow-up, there was no report of complicated infections such as pneumonia or sepsis.

## Introduction

We report a young male,who was admitted to the hospital because of worsening acute abdominal pain on the third day. Physical examinations showed tenderness and rebound pain at McBurney’s point in the lower right quadrant. Laboratory test results are as follows: WBC: 12.46×10^9^/L; PLT: 63×10^9^/L; neutrophils: 10.4×10^9^/L; blood amylase: 34 U/L; and urinary amylase: 327 U/L. An abdominal ultrasound showed abdominal organ inversion, splenomegaly, intestinal and lower abdominal effusion, and mesenteric lymph node enlargement. An abdominal computed tomography (CT) showed Abdominal organ inversion, Splenomegaly, and Intestinal effusion-labeled spleen.

## Case display

**Figure 1:**
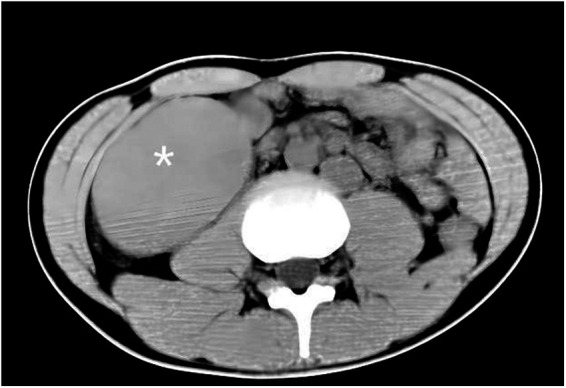
Coronal section CT scan of abdomen. Organ inversion, splenomegaly, and intestinal effusion-labeled spleen.

**Figure 2:**
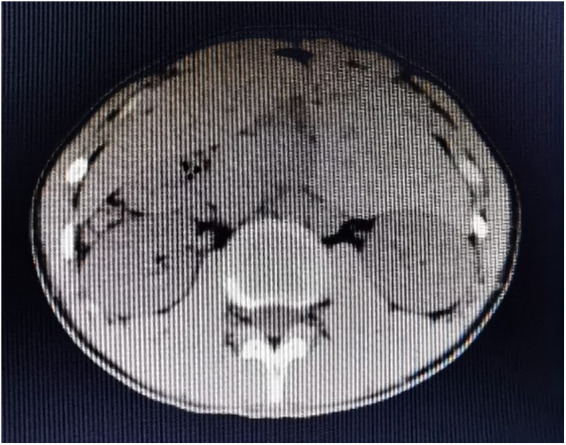
Abdominal CT reexamination on 24 November 2018. The spleen was absent.

The patient, an 18-year-old male, was admitted to the hospital on 24 November 2017 because of aggravation of acute abdominal pain on the third day and no fever, nausea, vomiting, and diarrhea on the first day. Abdominal physical examination showed that there was tenderness and rebound pain at McBurney’s point of the lower right abdomen. Also, there was no previous operation history. Laboratory test results are as follows: WBC: 12.46×10^9^/L; PLT: 63×10^9^/L neutrophils: 10.4×10^9^/L; blood amylase: 34 U/L; and urinary amylase: 327 U/L. He underwent urgent radiological examination, and the results were received in the shortest possible time. An abdominal ultrasound showed abdominal organ inversion, splenomegaly, intestinal and lower abdominal effusion, and mesenteric lymph node enlargement. An abdominal computed tomography (CT) showed abdominal organ inversion, splenomegaly, and intestinal effusion-labeled spleen (Figure 1). One year after the operation, a CT re-examination on 24 November 2018 showed that the spleen was absent, and no abnormality was found in other abdominal organs (Figure 2). Preoperative diagnosis showed abdominal organ inversion and considered acute appendicitis.

A laboratory review on 27 November 2017 showed the following: WBC: 9.02×10^9^/L; PLT: 191×10^9^/L; neutrophils: 6.4×10^9^/L; blood amylase: 34 U/L; and urinary amylase: 327 U/L.

**Figure 3:**
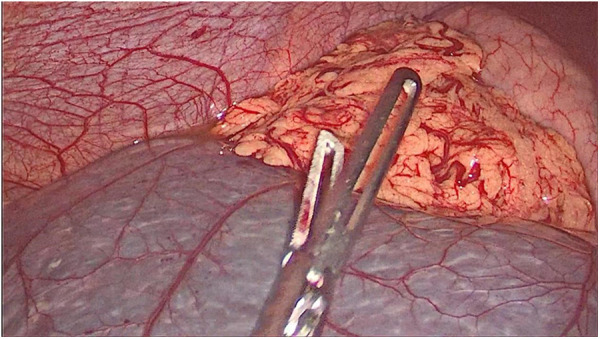
Intraoperative exploration of the spleen.

**Figure 4:**
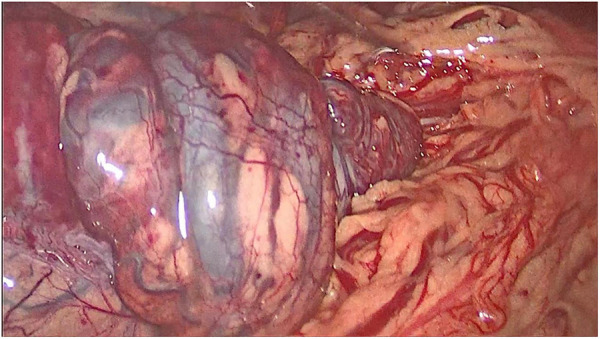
The twisted splenic pedicle.

**Figure 5:**
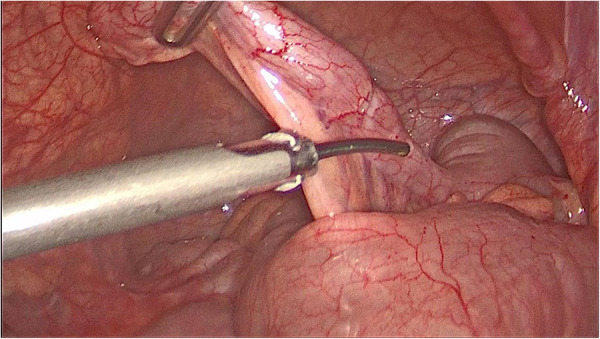
Dissection of the mesoappendix.

**Figure 6:**
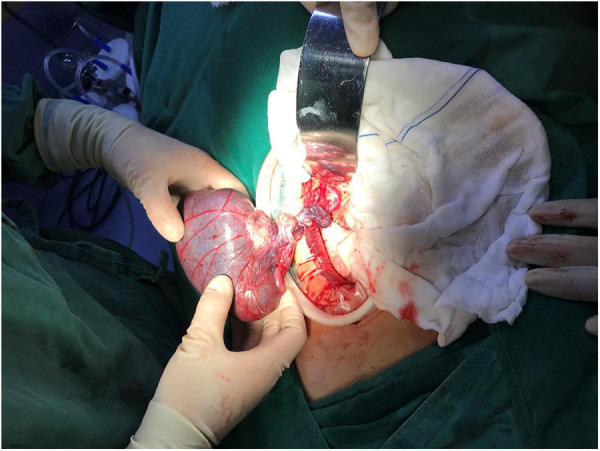
The resected infarcted spleen.

Abdominal laparoscopic exploration was performed after improving the preoperative examination and signing the operation consent. The exploration showed that the spleen was located on the right side of the abdominal cavity and covered with capsules on the surface (Figure 3), and the splenic pedicle was twisted (Figure 4). There was congestion in the appendix. After laparoscopic appendectomy (Figure 5), the infarcted spleen was removed under laparoscopic localization (Figure 6).

**Figure 7:**
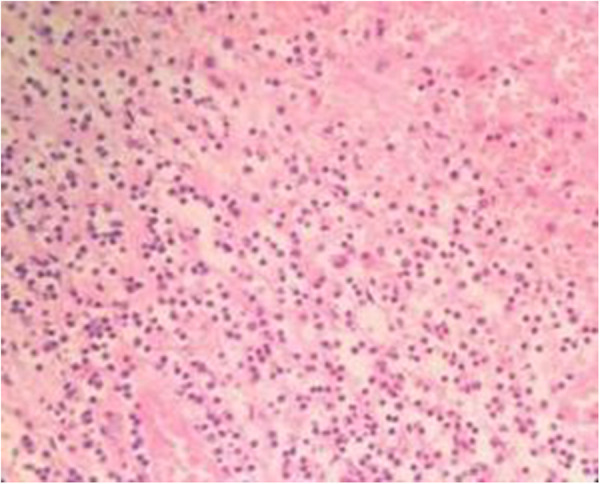
The pathology of the splenic pedicle (magnification × 400).

**Figure 8:**
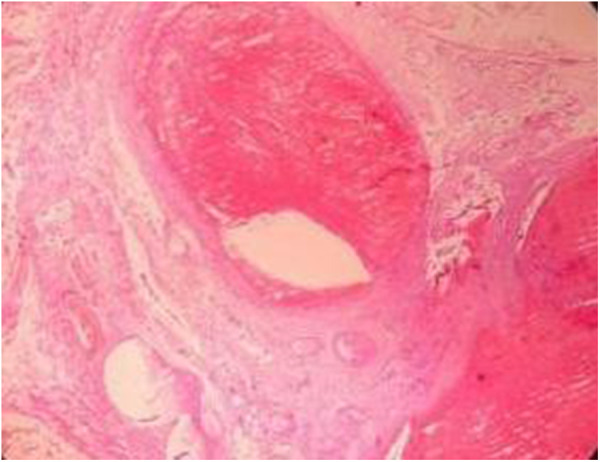
The appendix pathology (magnification × 100).

**Figure 9:**
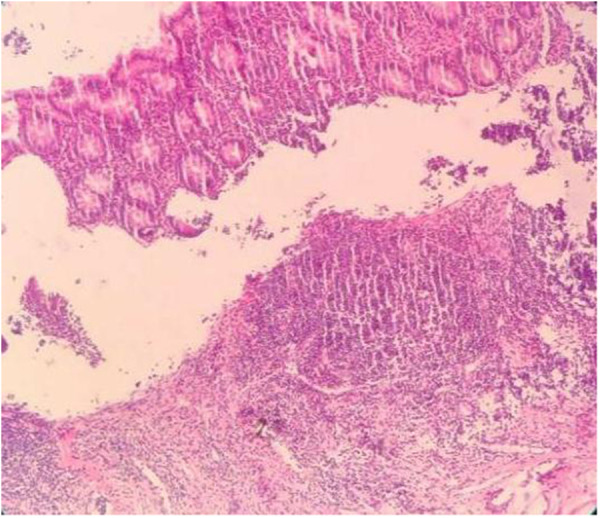
Inflammatory manifestation (magnification × 100).

Postoperative histopathological findings are as follows: spleen pathology (400×): significant congestion and bleeding in the splenic sinus, atrophy of the white pulp, and multiple focal infarcts under the touch that showed hemorrhagic infarct changes. Figure 7 shows the pathology of the splenic pedicle (400×): blood vessels were highly dilated and congested; some blood vessels were distorted; and the wall thickness was uneven, accompanied by edema, degeneration, and some thrombosis. Figure 8 shows the appendix pathology (100×). Figure 9 shows appendicitis.

The patient had a stable postoperative course with no abdominal bleeding and no peritonitis. The abdominal drainage tube was removed before discharge. His incision healed well, and his physical examination showed no abnormalities. At last, he was discharged 7 days after surgery.

## Discussion

Wandering spleen is characterized by the lack of normal peritoneal attachment. It can cause splenic ectopia due to the relaxation of ligaments in the spleen, kidney, and stomach (1). The spleen can then migrate to various positions in the abdomen or pelvis, which causes a complex clinical situation. Wandering spleen is a rare clinical disease. There is no specific age of onset. It is more common in women of childbearing age, which may be related to pregnancy (2). The clinical manifestations are variable, ranging from asymptomatic accidental discovery to acute abdomen complicated with torsion. Previous literature has reported that CT was the first imaging examination used (3–4).

This patient was admitted to the hospital with an acute abdomen. According to the results of color Doppler ultrasound and spiral CT, it was considered that the abdominal organs were reversed. The abdominal signs were tenderness and rebound pain in the right lower abdomen. Combined with the patient’s medical history, symptoms, and auxiliary examination, the diagnosis was acute appendicitis and abdominal organ inversion. Retrospective analysis, because the patient had tenderness and rebound pain at McBurney’s point of the lower right abdomen and elevated leukocyte levels, was consistent with the initial diagnosis of appendicitis. What is more, the patient’s pain suddenly worsened on the third day, which we speculate may have been caused by torsion of the wandering spleen.

There are two kinds of visceral inversion: full visceral inversion and partial visceral inversion with a totally reversed side. The latter has a population incidence rate of about 1 in 10,000 (the incidence rate is less than 1 in 1,000,000). Full visceral inversion is an extremely rare anatomical variation of human viscera. It refers to the 180° inversion of the position of all viscera, such as the heart, lung, diaphragm, liver, spleen, stomach, and intestine. Placement looks like a mirror image of normal organ placement, and the circulation, respiration, and digestion functions are normal. There is no conclusion on the cause of visceral inversion; it is mostly considered related to family genetics, and the distortion of the chromosome structure may be its basic cause.

Acute appendicitis leads to acute peritonitis, which is in line with the indication of emergency surgical treatment. Laparoscopic exploration of the abdominal cavity found that the anatomical position of abdominal organs was the same as that of unaffected people, and there was no organ inversion. At the same time, it was found spleen wandering and, near the lower right abdomen, splenic pedicle torsion and splenic infarction. The appendix was in the anterior position of the cecum with inflammatory changes. After a laparoscopic appendectomy, the infarcted spleen was removed under laparoscopic localization. Combined with the pathological results, the diagnosis was wandering spleen and appendicitis. Reflecting on the patient's diagnosis and treatment process, it can be seen that in the early stage of treatment, noninvasive imaging examination, ultrasound, and CT suggested that the patient had abdominal organ inversion. This is mainly because, on the one hand, wandering spleen is a rare clinical condition, the doctor lacked experience recognizing the condition, and the patient’s splenic pedicle torsion and splenic infarction differed from the morphological and imaging features of the spleen in the physiological state, which increased the difficulty of identification. On the other hand, the signs of abdominal infection caused by appendicitis interfered with the correct anatomical location. After the noninvasive imaging examination, the correct diagnosis could be made through laparoscopic exploration, and timely treatment could be provided.

Although the incorrect judgment of organ inversion does not affect the correct treatment of patients, the process of misdiagnosis offers us experiences worthy of reference. First, surgery is the first choice for the treatment of wandering spleen because conservative treatment will increase complications (5), and splenectomy or splenic fixation can be used for surgical treatment (6). Second, when the clinical manifestation is the acute abdomen, imaging diagnosis also suggests appendicitis and other types of abnormalities. We should not hastily attribute the cause of acute abdomen to appendicitis; instead, we should make further in-depth queries and carefully consider “other abnormalities,” including uncommon and rare situations, to improve the accuracy of prediction and make a better operation and nursing plan. Third, although imaging examination can provide valuable information, clinical doctors, especially surgeons, cannot overly rely on it. They should make an appropriate extension based on imaging diagnosis and formulate a backup plan when necessary to ensure that they know themselves and the enemy in the subsequent operation and do not fall into a passive situation. In addition, in the treatment of wandering spleen, there are risks of *Haemophilus influenzae*, pneumococcus, and *Neisseria meningitidis* after splenectomy and complications such as sepsis, increased cancer incidence, and higher thrombotic rates. Because of these risks, it is recommended that young patients retain their spleen for as long as possible (7). However, if splenic pedicle torsion causes splenic infarction, then a splenectomy is inevitable.

Although the patient was only 18 years old, he had splenic pedicle torsion and splenic infarction, which eliminated the possibility of preserving the spleen. Moreover, he had a long infarction time and a large ischemic area. Doctors did not rule out the formation of local thrombosis and microthrombosis. In this case, they should not have chosen splenic fixation because splenectomy was the most appropriate treatment. Through strengthening publicity and education and perioperative nursing, prophylactic use of antibiotics, the 23-valent pneumococcal polysaccharide vaccine, was conducted as planned 2 weeks after the operation, and there was no postoperative infection (8),nor did it increase the risk of long-term infection. The use of open or laparoscopic surgery depends on patients’ clinical status and doctors’ professional knowledge (9).

## Conclusion

This was a rare case of wandering spleen with splenic pedicle torsion. Appendicitis was present as well. The main cause of acute abdomen was wandering spleen with splenic pedicle torsion rather than appendicitis. Therefore, laparoscopic abdominal exploration was the first choice for the final diagnosis of wandering spleen, and splenectomy was the best treatment.

## Data Availability

The datasets presented in this article are not readily available because of ethical and privacy restrictions. Requests to access the datasets should be directed to the corresponding author.
