# Supplementary material for: A case report of wandering spleen with pedicle torsion and splenic infarction being misdiagnosed as organ inversion complicated with acute appendicitis
Source: Front Surg. 2022 Sep 6;9:916426. doi: 10.3389/fsurg.2022.916426 (PMC9485545; doi:10.3389/fsurg.2022.916426)
Supplement: Supplementary file 1 [file Data_Sheet_1_v1.pdf]

CARE case report guidelines

CARE Checklist of information to include when writing a case report

(cc) BY-NC-ND

✓

Topic	Item	Checklist item description	Reported on Line
**Title**	**1**	The diagnosis or intervention of primary focus followed by the words "case report"	
**Key Words**	**2**	2 to 5 key words that identify diagnoses or interventions in this case report, including "case report"	P1 L8
**Abstract (no references)**	**3a**	Introduction: What is unique about this case and what does it add to the scientific literature?	P1 L12
	**3b**	Main symptoms and/or important clinical findings	P1 L13
	**3c**	The main diagnoses, therapeutic interventions, and outcomes	P1 L12-16
	**3d**	Conclusion—What is the main "take-away" lesson(s) from this case?	P1 L12-17
**Introduction**	**4**	One or two paragraphs summarizing why this case is unique (**may include references**)	P2 L57-63
**Patient Information**	**5a**	De-identified patient specific information.	P2 L31
	**5b**	Primary concerns and symptoms of the patient.	P2 L31-34
	**5c**	Medical, family, and psycho-social history including relevant genetic information	P2 L34
	**5d**	Relevant past interventions with outcomes	NA
**Clinical Findings**	**6**	Describe significant physical examination (PE) and important clinical findings.	P2 L33-34
**Timeline**	**7**	Historical and current information from this episode of care organized as a timeline	P2 L45-55
**Diagnostic Assessment**	**8a**	Diagnostic testing (such as PE, laboratory testing, imaging, surveys).	P2 L33-42
	**8b**	Diagnostic challenges (such as access to testing, financial, or cultural)	NA
	**8c**	Diagnosis (including other diagnoses considered)	P3 L82-83
	**8d**	Prognosis (such as staging in oncology) where applicable	P3 L114-116
**Therapeutic Intervention**	**9a**	Types of therapeutic intervention (such as pharmacologic, surgical, preventive, self-care)	P2 L45-49
	**9b**	Administration of therapeutic intervention (such as dosage, strength, duration)	P2 L45-49
	**9c**	Changes in therapeutic intervention (with rationale)	P2 L39-44
**Follow-up and Outcomes**	**10a**	Clinician and patient-assessed outcomes (if available)	NA
	**10b**	Important follow-up diagnostic and other test results	P2 L50-55
	**10c**	Intervention adherence and tolerability (How was this assessed?)	NA
	**10d**	Adverse and unanticipated events	NA
**Discussion**	**11a**	A scientific discussion of the strengths AND limitations associated with this case report	P2-3 L57-92
	**11b**	Discussion of the relevant medical literature **with references**.	P2 L57-63
	**11c**	The scientific rationale for any conclusions (including assessment of possible causes)	P3 L107-118
	**11d**	The primary "take-away" lessons of this case report (without references) in a one paragraph conclusion	P3 L93-107
**Patient Perspective**	**12**	The patient should share their perspective in one to two paragraphs on the treatment(s) they received	NA
**Informed Consent**	**13**	Did the patient give informed consent? Please provide if requested	**Yes** ☑ **No** ☐
